# Three-dimensional prediction of nasal growth using landmark-based morphometry: Clinical validation of a predictive algorithm

**DOI:** 10.1016/j.jpra.2026.07.001

**Published:** 2026-07-07

**Authors:** Victor Pozzo, Marc-David Benjoar, Laura Charles, Golda Romano, Erik Zanchetta-Balint, Laurent A. Lantieri, Yael Berdah

**Affiliations:** a Department of Plastic, Reconstructive and Aesthetic Surgery, Hôpital Européen Georges Pompidou, Assistance Publique-Hôpitaux de Paris (APHP), Université Paris Cité, Paris, France; b Inserm UMR1272, Hypoxie et Poumon, Université Sorbonne Paris Nord, Faculté de Médecine SMBH, 93000 Bobigny, France

**Keywords:** Algorithm, Facial surgery, Nose, Innovation

## Abstract

**Background:**

Predicting nasal growth remains a major challenge in adolescent facial treatment. Current clinical decision-making relies primarily on chronological age and indirect skeletal maturity indicators, which poorly reflect individual nasal growth patterns. Three-dimensional facial imaging offers new possibilities for morphometric analysis; however, most existing growth models lack sufficient nasal resolution. This study aimed to clinically validate the geometric accuracy of a three-dimensional nasal growth prediction algorithm based on dense landmark-based morphometry.

**Methods:**

The predictive model was developed using a cross-sectional dataset of approximately 2050 multi-ethnic subjects aged 6–19 years with equal sex distribution. A total of 84 facial landmarks were collected, including 54 nasal-specific landmarks. A supervised multivariable linear regression model was trained on the standardised morphometric parameters, with age (continuous) and biological sex as covariates; no dimensionality reduction was applied. External validation was performed on a cohort of 12 longitudinally followed adolescents (6 female, 6 male; baseline 12.7 ± 2.6 years, follow-up 15.4 ± 2.7 years), strictly independent of the development dataset. The predicted post-pubertal morphology was compared with the observed follow-up acquisition using three complementary analyses: surface-to-surface deviation, Bland-Altman concordance on the Goode ratio and the nasal tip projection, and comparison against a no-growth baseline scenario.

**Results:**

The mean surface deviation between predicted and observed nasal morphology was 0.58 ± 0.20 mm (95% CI 0.46–0.70 mm; RMSE = 0.61 mm). 95.6 ± 5.0% of nasal surface points lay within the 1.5-mm clinical threshold and 99.5 ± 0.9% within 2.5 mm. Bland-Altman concordance analysis confirmed near-perfect proportional agreement on the Goode ratio (bias = −0.033; 95% limits of agreement [−0.145; +0.079]) and sub-threshold systematic bias on the nasal tip projection (bias = −1.01 mm, below the 1.5-mm clinical threshold; 95% limits of agreement [−4.10; +2.09] mm). The algorithm consistently outperformed the no-growth baseline scenario on both parameters, with the SD of the differences reduced by 12.3% and 8.7% respectively.

**Conclusion:**

The proposed three-dimensional algorithm provides geometric and morphometric validation of nasal growth prediction on a longitudinal adolescent cohort, with sub-millimetric global accuracy and concordant prediction on clinically relevant nasal parameters. These findings support the clinical applicability of the algorithm in the assessment of adolescent nasal growth, therapeutic planning and patient counselling, while accumulation of larger prospective datasets will continue to refine the precision boundaries across diverse populations.

**Level of evidence:**

Not applicable (Algorithm Validation Study)

## Introduction

Nasal growth plays a central role in facial harmony and continues later than most craniofacial structures, extending throughout childhood and adolescence. This prolonged and variable growth pattern makes surgical timing in pediatric and adolescent rhinoplasty particularly challenging. Premature intervention may interfere with residual growth, while delayed correction may prolong functional impairment and negatively affect psychosocial development. Despite these considerations, clinical decision-making remains largely empirical and experience-based.^1^, ^2^, ^3^

Current clinical practice relies primarily on chronological age, pubertal development, and indirect skeletal maturity markers, which remain poor predictors of individual nasal growth patterns.^4^, ^5^, ^6^ Consequently, predicting future nasal morphology remains one of the most debated issues in aesthetic nasal surgery, and reliable patient-specific tools to guide surgical timing are still lacking.^5^^,^^6^

The introduction of three-dimensional facial imaging has significantly improved morphological analysis and objective assessment of surgical outcomes.^7^, ^8^, ^9^ However, most existing three-dimensional growth models lack sufficient nasal resolution. This limitation primarily results from the limited number of facial landmarks and the absence of detailed representation of nasal subunits. Traditional anthropometric landmarks, although well established, are insufficient to accurately describe the complex geometry of the nose, particularly in a growing population.^10^, ^11^, ^12^ Recent studies in cranio-maxillofacial surgery have highlighted the potential of dense landmark-based three-dimensional modeling for facial growth analysis. However, nasal morphology remains poorly represented in most published models, and the geometric reliability of predictive approaches has rarely been validated before their potential clinical application.^13^, ^14^, ^15^

In this context, our team developed a three-dimensional nasal growth prediction algorithm specifically designed for clinical use. The purpose of the present study was to evaluate the geometric accuracy and morphological consistency of this algorithm through clinical validation in a pediatric and adolescent population.

## Materials and methods

### Study population and dataset construction

The predictive algorithm was developed using a cross-sectional dataset composed of approximately 2050 multi-ethnic subjects aged between 6 and 19 years, with an equal distribution of male and female participants.

All three-dimensional facial acquisitions were obtained at a single time point. No longitudinal follow-up was performed. The dataset therefore represents a theoretical population assembled specifically for algorithm development and validation purposes, rather than a prospective growth cohort.

#### Three-dimensional data acquisition

Standardized three-dimensional facial images were obtained under controlled acquisition conditions. Facial surfaces were processed to extract Cartesian coordinates (x, y, z) corresponding to predefined anatomical landmarks.

Data normalization procedures were applied to ensure inter-subject consistency and eliminate variations related to positioning, scale, and orientation.

#### Landmark definition

A total of 84 facial landmarks were collected for each subject. These included:

• 30 conventional craniofacial landmarks commonly described in the literature.
• 54 nasal-specific landmarks developed internally.

The nasal-specific landmarks were designed to overcome the limited spatial resolution of nasal landmarks previously reported in the literature and to enable detailed modelling of nasal subunits, including the dorsum, tip, alar rims, columella, and nasal base.

#### Morphometric analysis and feature extraction

From the extracted landmark coordinates, standardized three-dimensional nasal morphometric measurements were computed. These included linear distances, angular measurements, and proportional indices describing nasal projection, rotation, dorsal length, tip position, and overall nasal geometry.

Age was incorporated as a primary independent variable given its central role in craniofacial growth dynamics.

#### Machine learning model development

Using the complete set of morphometric parameters, a supervised machine learning model was trained to identify age-related growth patterns and infer nasal morphological evolution.

The algorithm was designed to predict future nasal morphology based on a patient’s baseline morphometric profile and chronological age. In practical terms, the model estimates post–growth peak nasal configuration from measurements obtained prior to peak growth velocity.

#### Algorithm validation

External validation was performed using a cohort of 12 patients with longitudinal three-dimensional facial acquisitions. The 12 longitudinal validation patients were strictly independent of the development dataset and did not contribute in any way to model training; the regression coefficients and the normalisation parameters were established solely on the development dataset, and validation acquisitions were subsequently processed through the identical preprocessing and normalisation pipeline without ever influencing model fitting.

All three-dimensional images and landmark-based measurements were obtained by a single experienced operator using the VECTRA® imaging system (Canfield Scientific, Parsippany, NJ, USA) to minimize inter-operator variability and ensure measurement consistency.

Patients included in the validation cohort had at least two standardized three-dimensional facial acquisitions performed during adolescence. The first acquisition was obtained before the individual peak growth period, and the second acquisition was performed after completion of the peak growth phase.

The timing of the peak growth period was assessed retrospectively based on anthropometric evolution between the two acquisitions, particularly increases in body height and weight, which were used as indirect indicators of pubertal growth acceleration.

For each patient, the validation procedure followed three steps:

A baseline three-dimensional facial acquisition was obtained prior to the peak growth period.

The algorithm generated a prediction of the expected nasal morphology after growth completion using baseline morphometric parameters and chronological age.

A second three-dimensional acquisition obtained after the peak growth period was used as the reference morphology.

### Sample size

No formal a priori statistical sample-size calculation was performed, because the study is a geometric and morphometric validation rather than a comparative clinical trial. The development dataset (∼2050 subjects) was assembled as a comprehensive sample designed to cover the entire 6–19-year age range with adequate density in every age and sex stratum. The longitudinal validation cohort (n = 12) is constrained by the rarity of standardised three-dimensional facial acquisitions obtained in the same adolescent both before and after the pubertal growth peak. On a post-hoc basis, the cohort provides a precision of ±0.13 mm on the mean surface deviation and a statistical power exceeding 99% to demonstrate that the prediction error is below the 1.5-mm clinical threshold, which is the primary objective of this validation.

### Predictive model

The predictive model is a supervised multivariable linear regression model, fitted by ordinary least squares. For each nasal morphometric parameter, the post-growth value is predicted from the patient's baseline morphometric profile, chronological age (treated as a continuous variable) and biological sex (included as an explicit predictor). No dimensionality reduction, latent embedding or automated feature-selection procedure was applied; the standardised morphometric parameters (linear distances, angles and proportional indices) were used directly as input features after normalisation. Ethnicity was deliberately not encoded as a separate predictor: no genetic ancestry testing was performed, and self-reported ethnicity is an unreliable biological descriptor in extensively admixed contemporary populations. Instead, the model was trained on a deliberately multi-ethnic, phenotypically diverse sample so that morphological variation associated with ancestry is captured continuously through the dense morphometric parameters themselves.

### Landmark reproducibility

Primary landmarking for the validation analysis was performed by an experienced study operator following a fixed, fully standardised anatomical protocol applied identically to every acquisition. Each of the 54 nasal-specific landmarks is defined by an explicit anatomical rule corresponding to an identifiable feature of a nasal subunit (dorsum, tip, alar rims, columella or base), rather than to an interpolated or geometrically ambiguous point. Inter-operator reproducibility was assessed by having a second independent experienced operator position all 84 nasal-specific landmarks on each of the 24 longitudinal three-dimensional scans of the validation cohort (12 patients × baseline and follow-up acquisitions); the two placements were performed independently and separated by at least one month. The intraclass correlation coefficient (ICC[2,1], absolute agreement, single measure) was computed on the 2016 paired coordinate observations for each spatial axis. The pooled ICC across the three spatial coordinates was 0.974 (95% CI 0.973–0.975), indicating excellent inter-operator reproducibility according to the classification of Cicchetti (1994).²¹ The per-axis ICCs were 0.991 (95% CI 0.990–0.992) for the lateral (x) coordinate, 0.723 (95% CI 0.702–0.743) for the vertical (y) coordinate, and 0.692 (95% CI 0.669–0.714) for the antero-posterior (z) coordinate. The high inter-operator concordance is consistent with published evidence of high reproducibility for comparable rule-based three-dimensional facial landmarking¹¹,¹² further supports the precision of the present approach.

### Statistical analysis

Concordance between predicted and observed nasal morphology was evaluated using a three-layer statistical framework. (i) Geometric accuracy was quantified as the mean surface-to-surface deviation between the algorithm prediction and the observed post-pubertal scan over the nasal region, summarised as mean ± SD with 95% confidence interval; an equivalence interpretation against a predefined clinically negligible margin of 1.5 mm^8,16^ was applied to the upper bound of the confidence interval of the mean. (ii) Morphometric concordance was assessed by Bland-Altman analysis on two prospectively selected nasal parameters of distinct nature, namely the Goode ratio (PRN–SN divided by N–PRN; Goode 1984), a dimensionless clinical surrogate of nasal tip projection, and the nasal tip projection (PRN–SN), a linear dimension central to rhinoplastic planning. For each parameter, the mean bias and the 95% limits of agreement (bias ± 1.96 × SD of the differences) were computed. (iii) A no-growth baseline comparator, corresponding to the hypothetical scenario in which baseline morphology remains unchanged through pubertal growth, was computed on the same parameters; algorithm performance was deemed superior to the baseline if both the absolute bias and the SD of the differences were reduced.

## Results

### Patient demographics and growth characteristics

A total of 12 patients with longitudinal three-dimensional facial acquisitions were included in the validation cohort. The cohort comprised 6 female and 6 male patients. Baseline demographic characteristics and growth parameters are summarized in Table 1.

**Table 1:** Baseline demographic and growth characteristics of the validation cohort (n = 12).

Variable	Value (*n=12*)
*Sex distribution*	6 Female (50%), 6 Male (50%)
*Age at baseline (years)*	12.7 ± 2.6
*Age at final follow-up (years)*	15.4 ± 2.7
*Weight at baseline (kg)*	42.8 ± 10.7
*Weight at final follow-up (kg)*	52.4 ± 10.6
*Weight change (kg)*	+9.7 ± 5.9
*Height at baseline (cm)*	153.3 ± 11.7
*Height at final follow-up (cm)*	165.7 ± 8.1
*Height change (cm)*	+12.3 ± 7.4
*Growth velocity (cm/year)*	4.6 ± 2.8

**Table 2:** Accuracy of algorithmic prediction based on surface deviation thresholds.

Patient	Mean Surface Deviation (mm)	Surface Points <1.5 mm (%)	Surface Points <2.5 mm (%)
P1	0.46	99.0	100
P2	0.50	100	100
P3	0.41	100	100
P4	0.76	86.2	97.8
P5	0.62	97.1	100
P6	0.32	100	100
P7	0.37	99.3	100
P8	0.85	94.3	100
P9	0.63	94.5	98.9
P10	0.45	97.6	100
P11	0.93	85.9	97.6
P12	0.73	93.7	100
***Mean ± SD***	0.58 ± 0.20	95.6 ± 5.0	99.5 ± 0.9
***RMSE***	**0.61**	—	—

RMSE = root mean square error. Mean surface deviation represents the average Euclidean distance between predicted and observed nasal surface points within the nasal region.

Surface-point percentages were recomputed as the exact arithmetic mean ± SD of per-patient values for numerical consistency. The original one-sample *t*-test against zero has been removed from the analysis (see Methods, Statistical Analysis).

Body weight and height increased between baseline and final follow-up acquisitions. Mean growth velocity during the observation period was **4.6 ± 2.8** **cm per year**.

### Accuracy of nasal growth prediction (Table 2)

**Fig. 1:**
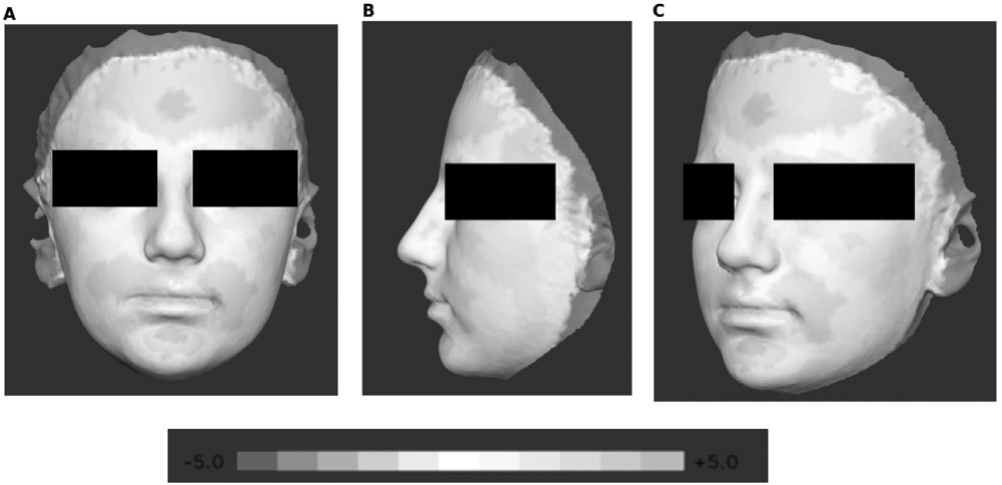
Three-dimensional surface superimposition between predicted and observed facial morphology. Representative frontal (A), lateral (B), and oblique (C) views showing superimposition of the predicted facial surface generated from the baseline scan and the observed facial scan obtained after growth completion in a representative patient. The baseline acquisition was performed at 13 years, and the follow-up scan at 16 years. Surface comparison was performed using the VECTRA® three-dimensional imaging system (Canfield Scientific, Parsippany, NJ, USA). The color scale represents surface deviation in millimeters, with white areas indicating minimal discrepancy (<1 mm). The colour scale represents the signed surface deviation (in mm) between the predicted and the observed nasal morphology, ranging from −5 mm (magenta, predicted surface posterior to observed) to +5 mm (cyan, predicted surface anterior to observed); the white range corresponds to near-zero deviations and the majority of the nasal surface appears within this range. Black bars have been applied over the orbital region (one over each eye, covering eye and eyebrow) to minimise patient identification; written informed consent for image publication was specifically obtained from the patient and the legal guardian, in accordance with ICMJE recommendations.

Quantitative surface analysis demonstrated high predictive accuracy of the algorithm. The mean surface deviation between predicted and observed nasal morphology was 0.58 ± 0.20 mm (95% CI, 0.46–0.70 mm; RMSE = 0.61 mm). Surface agreement remained high, with 95.6 ± 5.0% of nasal surface points within 1.5 mm, 99.5 ± 0.9% within 2.5 mm, and 61.8 ± 19.9% within 0.5 mm. The upper bound of the 95% confidence interval (0.70 mm) lies well below the predefined clinical equivalence margin of 1.5 mm,^8^^,^^16^ supporting clinical equivalence within the validation cohort. Surface superimposition between predicted and observed facial morphology is illustrated in Fig. 1. Representative frontal, lateral, and oblique views demonstrate the spatial distribution of signed surface deviations between the predicted nasal morphology and the follow-up facial acquisition obtained after the growth peak. The bidirectional colour scale (−5 mm magenta to +5 mm cyan; near-zero deviations in white) indicates the sign and magnitude of the deviation. Most regions appear within the white range, corresponding to deviations below 1 mm.

## Concordance analysis (Bland-Altman)

**Fig. 2:**
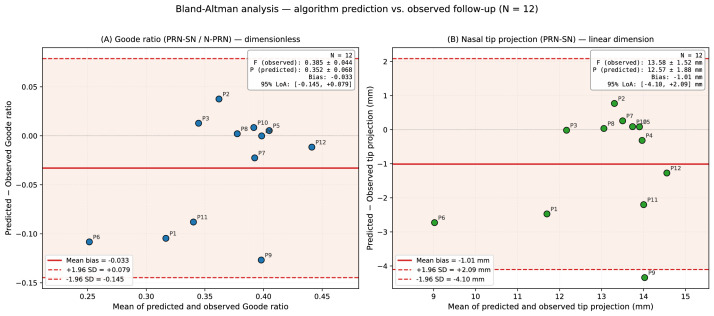
Bland-Altman analysis of the algorithm prediction against the observed post-pubertal morphology, performed on the 12 longitudinal validation patients. (A) Goode ratio (PRN–SN divided by N–PRN), a dimensionless clinical surrogate of nasal tip projection: mean bias −0.033, 95% limits of agreement [−0.145; +0.079]. (B) Nasal tip projection (PRN–SN), a linear dimension: mean bias −1.01 mm (below the 1.5-mm clinical threshold), 95% limits of agreement [−4.10; +2.09] mm. For each plot, the horizontal axis shows the mean of the predicted and observed values; the vertical axis shows their difference (predicted − observed). The solid red line indicates the mean bias; the dashed red lines indicate the 95% limits of agreement; the shaded band represents the 95% agreement region. PRN, pronasale; SN, subnasale; N, nasion.

Concordance between the algorithm prediction and the observed follow-up morphology was further evaluated by Bland-Altman analysis on two prospectively selected parameters (Fig. 2). On the Goode ratio (Fig. 2, panel A), the predicted value was 0.352 ± 0.068 versus 0.385 ± 0.044 observed (mean bias = −0.033, 95% limits of agreement = [−0.145; +0.079]; SD of differences = 0.057). On the nasal tip projection (Fig. 2, panel B), the predicted value was 12.57 ± 1.88 mm versus 13.58 ± 1.52 mm observed (mean bias = −1.01 mm, below the 1.5-mm clinical threshold; 95% limits of agreement = [−4.10; +2.09] mm; SD of differences = 1.58 mm). Compared with the no-growth baseline scenario, the algorithm prediction reduced the SD of the differences by 12.3% for the Goode ratio and by 8.7% for the nasal tip projection, with the absolute bias halved on the linear dimension (1.01 vs. 1.57 mm). The algorithm therefore reduces both the systematic bias and the variability of morphometric error compared with the no-growth baseline.

## Discussion

The present study demonstrates that the proposed three-dimensional nasal growth prediction algorithm achieves a high level of geometric accuracy when compared with longitudinal facial acquisitions obtained after the growth peak. Surface comparison analysis showed sub-millimetric discrepancies between predicted and observed nasal morphology, with a mean surface deviation of 0.58 mm and >95% of surface points remaining within 1.5 mm. In aesthetic nasal surgery, deviations below 1.5 mm are generally considered clinically negligible and fall within acceptable limits for morphological assessment and surgical planning.^8^^,^^16^ This validation should be regarded as a geometric and morphometric validation: it quantifies how closely the algorithm reproduces post-pubertal nasal morphology, and its clinical translation will benefit from continued data accumulation in larger and more diverse cohorts.

The high accuracy observed in the nasal region may be explained by the dense nasal landmark distribution incorporated into the modeling framework. While most previously published facial growth models rely on a limited number of nasal landmarks, the present approach integrates 54 nasal-specific landmarks designed to capture the fine geometry of nasal subunits, including the dorsum, tip, alar rims, columella, and nasal base. Previous studies in three-dimensional craniofacial morphometry have emphasized that insufficient landmark density represents a major limitation when modeling complex structures such as the nose.^10^, ^11^, ^12^, ^13^ By increasing the spatial resolution of nasal landmark sampling, the present algorithm may better capture subtle variations in nasal geometry during growth.

Beyond the nasal region, the algorithm also demonstrated good reconstruction of skeletal contours and osseous projections, consistent with findings reported in previous cranio-maxillofacial three-dimensional modeling studies.^13^, ^14^, ^15^ Minor discrepancies were observed in soft-tissue regions, particularly in the jugal area. These differences are likely related to variations in soft-tissue thickness and changes in body weight during adolescence, which can influence facial morphology independently of skeletal growth. Similar limitations have been described in previous three-dimensional facial growth analyses and represent a known constraint of cross-sectional modeling approaches.^11^^,^^14^

From a clinical perspective, predictive modeling of nasal morphology may represent a complementary tool for surgeons managing adolescent patients. Determining the optimal timing of treatment during facial growth remains challenging, and objective estimation of post-growth nasal morphology could contribute to surgical planning and patient counseling. Innovative treatments designed to medically control the nasal growth could be applied to patients at risk to develop proeminent nose. The increasing integration of three-dimensional imaging technologies into clinical practice further supports the potential applicability of such predictive frameworks.

The present validation, while focused on a single-centre longitudinal cohort, has been sufficient to demonstrate sub-millimetric geometric accuracy and excellent inter-operator reproducibility (pooled ICC 0.974) of the predictive algorithm. Three complementary directions for further development can be highlighted. First, expansion of the validation cohort to multi-centre patient populations will progressively characterise the generalisability of the algorithm across diverse clinical and demographic settings. Second, although the development dataset is cross-sectional, it captures population-level morphometric tendencies with sufficient fidelity to yield sub-millimetric predictions on a longitudinal validation cohort; subsequent enrichment with longitudinal training data will refine subject-specific prediction, particularly for atypical or extreme developmental trajectories that population-level models are less optimised to capture. Third, deployment of the standardised landmarking protocol to additional independent operators will further document its high transferability beyond the two operators evaluated here. These planned developments build directly on the present validation, which establishes the geometric and morphometric foundation supporting the clinical deployment of the algorithm in adolescent nasal growth assessment, therapeutic planning and patient counselling.

The algorithm consistently outperformed the naïve no-growth comparator and demonstrated proportional concordance with the observed post-pubertal morphology. Continued data collection in larger cohorts will further characterise per-parameter precision across diverse populations. These findings support the clinical applicability of the algorithm in the assessment of adolescent nasal growth, therapeutic planning and patient counselling, while accumulation of larger prospective datasets will continue to refine the precision boundaries across diverse populations.

## Ethical approval

This study complies with the Declaration of Helsinki. The development dataset consists of fully anonymized data obtained from an academic research database; because anonymized data fall outside the legal definition of personal data, this dataset did not require separate ethics-committee review. The longitudinal validation cohort comprised three-dimensional facial images acquired during routine clinical care. Under French law (loi Jardé), retrospective non-interventional studies based on data collected during routine care do not require approval from a Committee for the Protection of Persons. The study was conducted in compliance with the MR-004 reference methodology of the French Data Protection Authority (CNIL), to which our institution has declared conformity. Patients and their legal guardians were informed of the potential research use of the images and did not object. All data were anonymized prior to analysis and stored on secured institutional servers with restricted access. For the patient whose three-dimensional facial reconstruction appears in Fig. 1, written informed consent for image publication was specifically obtained from the patient and the legal guardian in accordance with ICMJE recommendations; as a precautionary measure, black bars have been applied over the orbital region (one over each eye, covering eye and eyebrow) of the published figure to further minimise patient identification.

## Funding

This research did not receive any specific grant from funding agencies in the public, commercial, or not-for-profit sectors.

## Declaration of competing interest

M-D. Benjoar and Y. Berdah have a financial/equity interest in Orthonose, a company active in nasal growth assessment and nasal orthopaedic technologies. M-D. Benjoar and Y. Berdah received no salary or payment from Orthonose for this work. Orthonose had no role in the study design, data collection, data analysis, interpretation, manuscript preparation, or decision to submit or publish this article. The remaining authors declare no competing interest.
